# Identification of a pathogenic founder variant in the *WFS1* gene that causes Wolfram syndrome in the Druze population

**DOI:** 10.3389/fped.2025.1525846

**Published:** 2025-01-29

**Authors:** Inbal Halabi, Yardena Tenenbaum-Rakover, Lena Sagi-Dain, Ilana Koren

**Affiliations:** ^1^ Pediatric Endocrine Unit, Carmel Medical Center, Haifa, Israel; ^2^ Clalit Health Services, Haifa, Israel; ^3^ Children’s Endocrinology Consulting Center, Clalit Health Services, Afula, Israel; ^4^ The Ruth & Bruce Rappaport Faculty of Medicine, Technion, Haifa, Israel; ^5^ Genetic Institute, Carmel Medical Center, Haifa, Israel

**Keywords:** Wolfram syndrome, diabetes mellitus, optic atrophy, endoplasmic reticulum, type 1 diabetes mellitus, diabetic ketoacidosis

## Abstract

**Context:**

Wolfram syndrome (WS) is an autosomal recessive neurodegenerative disorder caused by pathogenic variants in the *WFS1* gene. It is characterized by central diabetes insipidus, juvenile-onset diabetes mellitus (DM), optic atrophy (OA), and deafness. The natural history of WS is variable, even within the same family and with the same variant.

**Objective:**

To report the phenotypes of five patients of Druze origin, all carrying the same autosomal recessive pathogenic variant in the *WFS1* gene.

**Patients & methods:**

Five patients belonging to three core families were enrolled. Clinical, biochemical, and genetic data were retrieved retrospectively from their medical files.

**Results:**

All five patients carried the same previously reported homozygous *WFS1* pathogenic variant: c.2649del, p.Phe884fs. In all patients, the first presentation was DM at a mean age of 5.2 years (range 4–7), diagnosed initially as type 1 DM with negative anti-pancreatic autoantibodies, and all were treated with insulin by either pump or multiple injections. All five patients had OA that appeared at a mean age of 12.3 years (range 4–30). Three had hearing loss and neurological involvement, and none had diabetes insipidus. One patient was treated with a glucagon-like peptide 1 receptor agonist with a good response.

**Conclusions:**

This is the first report of a founder pathogenic variant in the *WFS1* gene in the Druze population in Israel. Our findings imply that molecular analysis is warranted in children presenting with DM and negative pancreatic antibodies. The identified variant should be considered for genetic testing in individuals of Druze ancestry diagnosed with young-onset non-autoimmune diabetes. Early diagnosis of WS is important for therapeutic approaches, especially since novel medications are becoming available.

## 1 Introduction

Wolfram syndrome (WS, OMIM: 222300) (1), is a rare autosomal recessive neurodegenerative disorder characterized by central diabetes insipidus, juvenile-onset diabetes mellitus (DM), optic atrophy (OA), and deafness—also referred to as DIDMOAD (2). The disease was first described by Wolfram and Wagener in 1938 in four siblings with the coexistence of juvenile-onset DM and OA (3). The prevalence of WS has been estimated to vary between 1:770,000 people in the United Kingdom and 1:100,000 in North America (4). A higher incidence has been reported in consanguineous populations, e.g., Lebanese and Sicilian populations (5, 6). Most patients with WS carry biallelic pathogenic variants in the Wolframin, a transmembrane glycoprotein gene (*WFS1*), located on human chromosome 4p16.1, which encodes the 890-amino-acid endoplasmic reticulum (ER)-membrane-located protein wolframin (7, 8). A subgroup of patients has been reported with dominant *WFS1* pathogenic variants, which result in a milder phenotype characterized by the clinical triad of congenital progressive hearing impairment, DM, and OA (OMIM 614296) (7–10). A smaller proportion of patients have pathogenic variants in the *CIDS2* gene (encoding CDGSH iron-sulfur domain-containing protein 2), resulting in an autosomal recessive type of WS termed Wolfram syndrome 2 (7).

*WFS1* encodes wolframin, a protein which is highly expressed in the brain, pancreatic *β*-cells, heart, lungs, and placenta (7). It plays an important role in Ca^2+^ homeostasis and it is important for maintaining endoplasmic reticulum (ER) stress response via tight regulation of stress signaling through its interaction with a key transcription factor, ATF6*α*, thereby protecting cells from the damaging effects of this signaling pathway's hyperactivation (9, 11). The ER is a cellular organelle responsible for the storage of Ca^2+^ ions, and for the correct folding and post-translational modification of several proteins. It has been proposed that because wolframin has a protective function against ER stress, biallelic loss-of-function pathogenic variants in *WFS1* gene cause an increase in the cytosolic concentration of Ca^2+^ ions, resulting in the establishment of chronic ER stress and the inappropriate activation of the unfolded protein response signaling pathway. These events lead to an unresolvable high level of ER stress that causes *β*-cell death and neurodegeneration (8, 11, 12). Furthermore, studies have demonstrated that WFS1 plays a crucial role in regulating the dynamic interaction between mitochondria and mitochondria-associated membranes (MAMs). These MAMs act as close contact domains with the ER, facilitating the transport of Ca^2+^ from the ER to mitochondria, thereby influencing mitochondrial function (8). A deficiency in Wolframin leads to the disorganization of MAMs, resulting in reduced mitochondrial Ca^2+^ uptake (8). A common presenting symptom of WS is non-autoimmune insulin-dependent DM developing in the first decade of life. Therefore, many patients affected by WS may initially be misdiagnosed as type 1 DM (T1DM). In contrast to T1DM, however, patients with WS present without diabetic ketoacidosis (DKA) at onset, the remission period is longer, and insulin requirement and glycated hemoglobin (HbA1c) levels are lower (13). OA occurs in the first decade of life; it begins with reduced visual acuity and loss of color vision and may lead to blindness (13). Hearing impairment is usually diagnosed in the second or third decade of life at an average age of 12.5 years (range, 5–39) and manifests in 62% of patients with WS, disturbing firstly high frequencies and progressing relatively slowly (7, 13). With increasing age, hearing impairment is more pronounced than in other types of hearing loss, probably because of the progressive central nervous system degradation process (7). In addition to the main manifestations of the disease, patients may exhibit various urological and neuropsychiatric symptoms (14). About 62% of patients have neurological complications, appearing between 5 and 44 years of age (7, 13, 14). The most common neurological symptom is cerebellar ataxia of the trunk, and other signs include dysarthria, dysphagia, areflexia, epilepsy, nystagmus, and headaches (7, 13). Brain magnetic resonance imaging (MRI) abnormalities include cerebellar and brain stem atrophy, and cortical atrophy (5). Psychiatric manifestations include anxiety, panic attacks, and mood swings (13). Urological problems have been reported in 19% of WS patients, including upper urinary tract dilatation, urinary incontinence, and recurrent infections related to neurogenic bladder appearing at around the age of 20 years (14). Some patients may die prematurely in the third decade of life due to central respiratory failure (14). Due to its molecular complexity, it is difficult to establish a clear association between genotype and phenotype (7, 13, 14). Recently, a mild late-onset phenotype of WS was reported in Ashkenazi Jews in Israel, caused by the founder mutation (c.1672C>T, p.R558C) in the *WFS1* gene. This pathogenic missense variant was identified in 1.34% of the Ashkenazi Jewish population in Israel (15). In the present study, we report on a founder pathogenic variant in the *WFS1* gene in five patients belonging to three core families of Druze origin.

## 2 Case descriptions and results

The study was approved by the Carmel Medical Center Institutional Review Board (approval no. 0116-20-CMC, year of approval 2020). The clinical characteristics of the five patients are summarized in Table 1. and their diabetes characteristics in Table 2.

**Table 1:** Summary of patients’ clinical characteristics.

Patient no.	Sex	Family no.	Current age (Years)	Age of DM onset (Years)	Age at OA diagnosis (Years)	Diabetes insipidus	Age at hearing loss diagnosis (Years)	Neurological involvement	Urological involvement
1	M	1	7.4	5.1	5.6	–	5.9	Headaches	–
2	M	2	25.1	7	14	–	–	Headaches	–
3	F	2	Died^a^	4	4	–	23	–	+ (kidney involvement)
4	F	3	12.1	4	8	–	–	–	–
5	F	3	34	6	30	–	–	Mood lability	Severe bilateral hydronephrosis, urine retention
Mean			19.7 (7.4–34)	5.2 (4–7)	12.3 (4–30)		14.5		
Total	2M/3F	3		5/5	5/5	0/5	2/5	3/5	2/5

^a^ Died at the age of 27 years from WS complications.

**Table 2:** Clinical characteristics of diabetes mellitus.

At diagnosis					At follow-up				
Patient no.	DKA	C-peptide (pmol/L)	HbA1c (%)	GADA/ICA	Hypoglycemic episodes	Total insulin units (kg/d)	HbA1c (%)	Mode of therapy	Duration of the disease at the time of study (Years)
1	No	340	9.6	Negative	Yes	0.3	6–7	CSII + GLP1-RA	2.3
2	No	874	6.2	Negative	Yes	0.3	9–11	MDI + CSII	18.1
3	No	NA	NA	Negative	NA			MDI	23
4	No	884	6.9	Negative	Yes	0.45	6.5–7.5	MDI	8.1
5	No	700	7	Negative	Yes	0.8	9–10	MDI	28
Normal range		360–1,660							

DKA, diabetic ketoacidosis; HbA1c, glycated hemoglobin; GADA/ICA, glutamic acid decarboxylase autoantibodies/islet cell cytoplasmic autoantibodies; NA, not applicable; CSII, continuous subcutaneous insulin infusion; GLP1-RA, glucagon-like peptide-1 receptor agonist; MDI, multiple daily injections.

### 2.1 Case 1

A male born to healthy first-degree cousins of Druze origin was referred at the age of 5.10 years to our diabetic clinic with a diagnosis of T1DM made at the age of 5.1 years, presenting without DKA. Family history revealed a paternal non-obese uncle with type 2 DM (T2DM) diagnosed at the age of 28 years. Treatment was initiated with a rapid-acting insulin analog (insulin aspart, NovoRapid) via a continuous subcutaneous insulin infusion (CSII) pump. Shortly after the diagnosis of T1DM, OA was detected and a few months later, sensorineural hearing loss was diagnosed. Pancreatic autoantibodies were all negative [anti-islet cell, anti-glutamic acid decarboxylase (GAD), and anti-insulin antibodies]. At the presentation, his body mass index (BMI) was in the 60th percentile. Brain MRI and ultrasound imaging of the urinary tract were without abnormal findings. Despite a low total daily insulin dose of 0.3 U/kg (HbA1c of 7%), he had several episodes of hypoglycemia, mainly at night. Serum and urinary osmolarity were within the normal range, excluding diabetes insipidus. Celiac serology and anti-thyroid antibodies were negative. Next-generation sequencing (NGS) panel for monogenic diabetes that included 117 genes was performed and revealed a homozygous pathogenic variant in *WFS1* gene, c.2649del, p.Phe884fs. Sanger sequencing of family members found both parents and the elder brother being heterozygous carriers of the identified pathogenic variant. At the age of seven years, following the diagnosis of WS, liraglutide (Victosa) was initiated at a dose of 0.3 mg/day subcutaneously, and the insulin dose was reduced by 40% in the pump. No adverse events were reported.

### 2.2 Case 2

A 25.1-year-old male born to healthy first-degree cousins of Druze origin presented with DM at the age of seven years. He was treated first with multiple daily injections (MDI) of insulin (rapid-acting insulin analog before each meal and long-acting analog once daily), and then switched to a CSII pump with rapid-acting insulin analog (NovoRapid). On this therapy, he had HbA1c within 9%–11%. At the age of 14 years, bilateral OA was diagnosed without evidence of diabetic retinopathy. Anti-GAD and anti-insulin autoantibodies were negative, as were anti-thyroid antibodies and celiac serology. A brain MRI performed at the age of 17 years following complaints of chronic headaches was without any pathological findings. Whole exome sequencing (WES) was performed and identified the same homozygous pathogenic variant as in case 1—c.2649del, p.Phe884fs. The parents were heterozygous carriers. Further family history revealed that the proband had a 4-year-old sister with DM (case 3).

### 2.3 Case 3

The sister of patient 2 was diagnosed with T1DM at the age of four years and was treated with MDI of insulin. She was diagnosed with OA at the the same time which progressed to blindness. In addition, she exhibited kidney involvement and cardiovascular diseases. Sensorineural hearing loss was found at the age of 23 years. Sanger sequencing revealed the same homozygous mutation as her brother—c.2649del, p.Phe884fs. She died at the age of 27 years from a sudden myocardial infarction.

### 2.4 Case 4

A 12.1-year-old female born to healthy Druze parents with remote family relations. She was diagnosed with T1DM at the age of four years. She was treated with MDI of rapid-acting insulin twice daily before meals and long-acting analog once daily, for a total daily insulin dose of 0.45 U/kg. HbA1c was 7.1%, and no episodes of DKA were reported. She had a long period of low insulin requirement after the onset of DM. Anti-GAD and anti-insulin autoantibodies were negative, as were anti-thyroid antibodies and celiac serology. At the age of 8 years, due to gradual blurring of vision, an eye examination revealed bilateral optic disk pallor with no signs of diabetic retinopathy. The visual field test demonstrated peripheral field reduction. Her BMI was in the 40.5th percentile. Serum and urine osmolarity were normal. Brain MRI at the age of nine years revealed atrophic changes of the optic nerves with no other anomalies. WES was performed and identified the same homozygous pathogenic variant—c.2649del, p.Phe884fs—as the other patients. The patient had a 34-year-old sister with T1DM (case 5).

### 2.5 Case 5

A 34-year-old woman with two healthy children had been diagnosed with T1DM since the age of 6 years and was treated with MDI of rapid-acting insulin before each meal and long-acting analog once a day for a total daily insulin dose of 0.8 U/kg. Her HbA1c ranged within 9%–10%. Anti-GAD and anti-insulin autoantibodies were negative, as were anti-thyroid antibodies and celiac serology. During follow-up, she exhibited several hypoglycemic episodes with no episode of DKA. She suffered from reduced visual acuity and loss of color vision for more than a decade, and at the age of 30 years, was diagnosed with bilateral OA. At the age of 28 years, severe bilateral hydronephrosis and urine retention and a neurogenic bladder with high post-void residual volume were diagnosed by ultrasound imaging, necessitating intermittent self-catheterization. She exhibited mood fluctuations and behavioral problems. At the age of 30 years, Sanger sequencing identified the same homozygous mutation as her sister (case 4)—c.2649del, p.Phe884fs.

### 2.6 Molecular analysis

**Figure 1:**
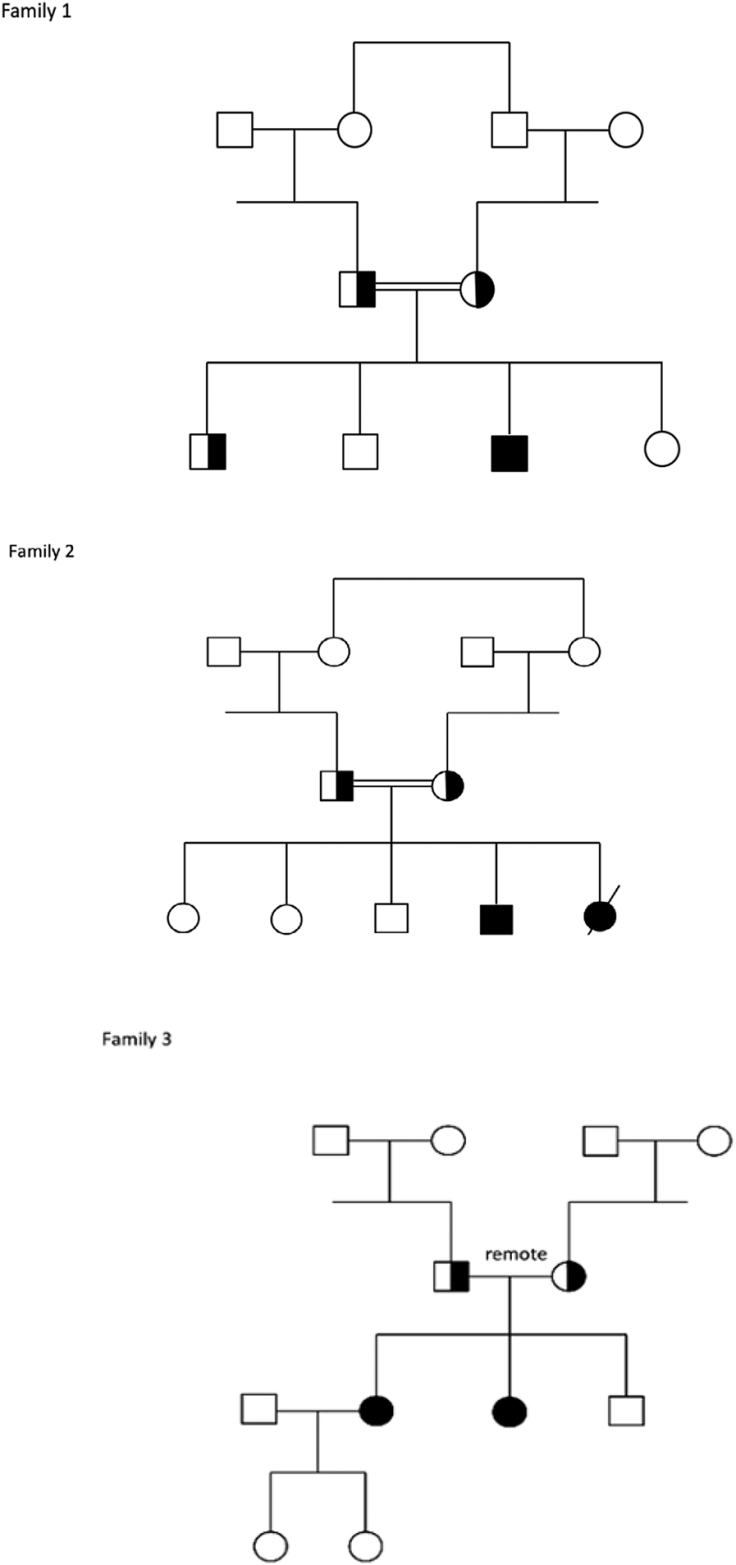
Families pedigrees.

The five reported patients shown in Figure 1, all of Druze origin and belonging to three different families living in remote villages in the northern region of Israel, carried the same variant, NM_006005.3(WFS1):c.2649del, p.Phe884Serfs*68 (chr4-6304170-TC-T) in a homozygous state. The variant is located in the last (8th) exon of the WFS1 gene, seven amino acids prior to the stop codon, causing a frameshift predicted to extend the protein by 60 additional amino acids, with most loss-of-function variants in WFS1 classified as pathogenic/likely pathogenic (cite Clinvar). In Addition, this variant is not found in the healthy population database (gnomAD) and has been previously reported in patients with WS (16). Therefore, the variant was classified according to American College of Medical Genetics and Genomics (ACMG) guidelines as pathogenic.

To confirm the pathogenic variant detected by NGS (monogenic diabetes panel and WES) and to perform family segregation analysis, Sanger sequencing was performed. Standard polymerase chain reaction (PCR) was carried on index case's and family members' DNA by using the forward 5'- AGTTCAGCACCATCCTGGAG -3' and reverse 5' – TACACAGCAGCCTTCCCTTT -3′ primer pair in the exon 8 of WFS1 gene. PCR products were analyzed by standard Sanger dideoxy nucleotide sequencing using 3500XL Genetic Analyzer (Thermo Fisher Scientific).

## 3 Discussion

We report on five patients homozygous for the same pathogenic variant in *WFS1*, all of Druze origin, presenting with a variable clinical phenotype of WS. This study presents the first documented founder pathogenic variant of *WFS1* in the Druze population in Israel. These patients live in different geographical areas in the northern region of the country. Most Druze live in villages in the Galilee and Golan Heights. They are a religious–ethnic group that marry only among themselves and often practice consanguinity. Founder pathogenic variants in monogenic disorders are expected in this community and indeed, unique nonsense pathogenic variants in the low-density lipoprotein receptor gene in Druze familial hypercholesterolemia pedigrees have been previously reported (17).

WS is a progressive neurodegenerative disorder in which patients typically develop DM in the first decade of life, followed by OA (2, 18). All patients in our cohort were initially misdiagnosed as T1DM with disease onset between 4 and 7 years of age. However, none of them were presented with DKA and all were negative for pancreatic autoantibodies (Table 2). All had a longer duration of remission, lower insulin requirements, and lower HbA1c levels than expected in patients with T1DM. The slower disease progression could be related to either greater pancreatic *β*-cell reserves or better insulin sensitivity (19); however, after long disease duration, as was demonstrated in patients 2 and 5, HbA1c levels increased, most probably due to progressive degeneration of pancreatic *β*-cells, where WFS1 is highly expressed. The finding of negative pancreatic autoantibodies, as well as the low daily insulin requirement in DM, led us to suspect monogenic diabetes.

All five patients had reduced visual acuity with no evidence of diabetic retinopathy; first, peripheral narrowing of the visual field developed, and then, OA occurred (20). OA is a major clinical presentation of WS and is characterized by a progressive decrease in visual acuity and color vision deficiency, often leading to blindness (7, 13). The onset of visual failure varies across different hereditary OA types. In WS, vision loss begins in early childhood, and typically, the rate of visual deterioration is faster compared to other OA types (8). Identifying pathogenic variants in specific populations in Israel facilitates prenatal diagnosis, thereby preventing OA in future generations (21). Only one patient in our cohort developed blindness; however, the patients are still young, and OA may progress with time. Annual eye examinations are recommended, including examination of visual acuity, color vision fundoscopy, visual field, and optical coherence tomography scan (13). The pathogenesis of OA might result from the effects of the absence of wolframin in the retinal ganglion cells (18).

None of the patients had DI, contrary to a previous report of approximately 38% of patients with WS exhibiting DI in the second decade of life (14). The first patient in this report developed DM at the age of 5.1 years, and OA at nearly the same age, earlier than has been reported in other cases (2, 18). It has been postulated that the age of onset of DM and OA is dependent on the production of WFS1 protein, which is related to the severity of the specific *WFS1* pathogenic variant (14). The c.2649del, p.Phe884fs mutation is located in the last (8th) exon of *WFS1,* resulting in a stop codon. All of our patients had severe phenotypes and presented early in life, suggesting a severe pathogenic variant compared to the relatively mild late-onset *WSF1* pathogenic variant described in Ashkenazi Jews in Israel (15). Our findings are consistent with the results of a previous study that demonstrated a dose-effect relationship between the severity of the variants, showing that nonsense and frameshift variants led to earlier onset of DM and OA compared to missense variants, indicating a strong genotype-phenotype correlation (9). This homozygous pathogenic variant was previously reported in two members of the same family from Denmark, both presenting with early-onset DM and OA (16). Additionally, it was identified in a 48-year-old man with depression and myocardial infarction who was compound heterozygous for the reported frameshift variant and the nonsense variant c.1999C > T (22).

Patient 3 died at the age of 27 years from a sudden myocardial infarction. Reports on cardiovascular involvement in WS are scarce. Indeed, cardiovascular autonomic neuropathy is a well-known complication of long-lasting diabetes. It has been reported that 16.1% of patients with WS exhibit valvular heart disease, and pulmonary stenosis (5). In our cohort, the heterozygous carriers were healthy and did not exhibit any clinical features of WS.

Due to high rates of consanguinity in the Druze population, additional patients diagnosed with either T1DM or type 2 DM might carry the same founder pathogenic variant. Raising family physicians' and pediatricians' awareness of the phenotypes of WS is important, to avoid erroneous diagnosis of T1DM and to provide genetic consultation for families (13, 23, 24). The combination of early-onset DM with negative pancreatic autoantibodies and OA should raise suspicion of WS, warranting molecular analysis. We used NGS panel which includes 117 genes for screening for monogenic diabetes and until now no founder pathogenic variants were identified in the Druze population in Israel**.** Although there is currently no specific treatment for WS, a number of complementary therapeutic strategies are being explored, including drug repurposing and gene therapy (8), emphasizing the importance of prompt and early diagnosis that may improve the treatment modality and slow the disease progression. The hallmarks in new therapies for WS are targeting ER stress to modulate it, regulating ER calcium homeostasis, and cellular proteostasis (25). Some of these therapies include chemical chaperones, ER calcium stabilizers, mitochondrial modulators, and GLP1-RAs (25). Recent studies have shown evidence for a beneficial effect of GLP1-RAs on WFS1-deficient human pancreatic *β*-cells and neurons, suggesting their use as a treatment for individuals with WS. GLP1-RAs have been shown to decrease pancreatic *β*-cell apoptosis mediated by ER stress, promote *β*-cell growth and survival, improve mitochondrial function, and reduce oxidative stress (12, 26, 27). Frontino et al. (28) provided the first preliminary report on the use of daily liraglutide (GLP1-RA) for 8–27 months and demonstrated its safety, tolerability, and efficacy in four pediatric WS patients aged 10–14 years. It has been established that GLP1-RAs improve glycemic control in both rodents and patients with WS by *β*-cell adaptation and preventing their apoptosis (12, 28). GLP1 is an incretin hormone, and its main action is to improve glycemic control by stimulating glucose-dependent insulin secretion and promoting insulin synthesis. According to Schäfer et al. (29), there is an association between genetic variations in the *WFS1* locus and reduced GLP1-induced insulin secretion which lead to a higher risk of type 2 DM. Thus, alterations of ER homeostasis derived from *WFS1* variants may be associated with impaired incretin action and, consequently, *β*-cell dysfunction. Therefore, it can be assumed that activation of the GLP1 receptor signal using GLP1-RAs will restore the incretin deficiency, alleviating insulin insufficiency and improving glycemic control (12). Bendotti et al. (30) reported that GLP1-RAs can modulate the immune system in both mice and humans, independently of the weight loss or glycemic state of the subject. Jagomäe et al. (31) suggested that treatment with GLP1-RAs can delay the onset of diabetes and protect against the development of OA and vision loss in a WS rat model. Current data indicate GLP1-RAs' effective use as therapeutic agents in WS, by improving glycemic control, and they are promising candidates for delaying neuronal-related symptoms in these patients (12). In this report, patient 1 received liraglutide therapy at seven years; pump insulin requirement was reduced, and improved glycemic control was observed by better time in range and reduction in HbA1c. Recent studies highlight the critical role of WFS1 in maintaining mitochondrial function and the consequences of its dysfunction (32–35). WFS1 is highly enriched at mitochondria-associated membranes (MAMs) and plays an integral role in MAM architecture, positioning it as a key regulator of mitochondrial function. Inherited pathogenic variants in *WFS1* typically result in decreased protein stability, altering its homeostasis and reducing ER to mitochondria calcium ion transfer, leading to mitochondrial dysfunction and cell death (32–35). Zatyka et al. (36) provided evidence of the relationship between WFS1 depletion and mitochondrial impairment in cortical neurons derived from patients with WS. Furthermore, pharmacological strategies that enhance ER Ca^2+^ uptake or prevent its leakage have been shown to restore normal cellular functions (37). Notably, activating the sigma-1 receptor, which is involved in calcium transfer, could potentially rectify MAM function compromised by wolframin deficiency (34). These recent findings underscore the potential for genetic and pharmacological interventions as therapeutic strategies to address mitochondrial dysfunction in WS, offering hope for improved patient outcomes.

## 4 Conclusions

We report on a pathogenic founder variant in *WFS1* causing WS in five patients of Druze origin, with variable phenotypes. Molecular analysis for monogenic diabetes is warranted in children with DM and negative pancreatic autoantibodies mainly in consanguineous communities. Early diagnosis of WS may change the management of DM, leading to close monitoring of additional systemic complications and to early therapeutic intervention. Novel promising therapies are to be considered to preserve *β*-cell function and reduce the progression of OA and neurological deterioration.

## Data Availability

The original contributions presented in the study are included in the article/Supplementary Material, further inquiries can be directed to the corresponding author.
